# Extracts from the Records of the Boston Society for Medical Improvement

**Published:** 1858-04

**Authors:** F. E. Oliver

**Affiliations:** Secretary


*Extracts From the Records of The Boston Society for Medical Improvement.* By F. E. OLIVER, M. D., *Secretary.*

*Tubular Pregnancy.*—In reference to the case mentioned in the last report of the Society, by Dr. HOOKER, Dr. H. stated that he was guided in his diagnosis by the case of tubular pregnancy reported in April by Dr. JACKSON, and which occurred under the care of Dr. Brooks, of the State Alms-House at Monson—(See *Journal,* Vol. LVI., p. 378).

It will be seen in the cases subsequently referred to by Dr. Jackson, and following that report, that this is the second instance in which the practitioner was materially aided in arriving at a correct diagnosis, by the previous report of a similar case; a fact illustrative, as Dr. Jackson remarks, of the practical importance and benefit to the profession, of medical associations.

DEC. 14th.—*Bronzed Skin; Atrophy of the Supra-renal Capsules.*—Dr. HODGES said that a subject had been received for the dissecting-room, from one of the public institutions, which presented a very striking discoloration of the skin, at once suggesting the melasma Addisonii.—The fore arms and the genitals were the most strongly marked. The spots were irregular but distinct in outline, reminding one of the changes sometimes observed in the negro. The skin of the face, legs and abdomen was also discolored, but in a less degree. The surface of the skin was natural, not having a scaly or furfuraceous appearance. There was an old reducible hernia on the right side.

On examination, no disease was found, to account for his death —The kidneys were healthy; the capsules of half their normal size, the right weighing 36, and the left 44, grains. Their appearance was certainly not that usually noticed in dissecting-room subjects; although it cannot be said that they were diseased.

With regard to the previous history of the person, Dr. H. stated that he was from the country, and aged about 75. He was registered in the books of the institution as "insane." He was never admitted to the Hospital, as he complained of nothing but debility; but was put into the " old man's" ward, so called, and allowed to lie in bed when so inclined. He died, suddenly, at the end of one week after his admission to the institution.

Dr. H. remarked that debility and cerebral disturbance were the two most marked symptoms of bronze disease, and regretted that the symptoms of death in this case were not recorded.

DEC. 28th.—*Bronzed Skin; the Supra-renal Capsules apparently*

*normal.*—Dr. HODGES showed the skin of the scrotum, the supra-renal capsules, and portions of the peritoneum, from a dissecting-room subject. The case was one of discoloration of the skin, similar to that above reported by Dr. H. The discoloration was not so marked as in the previous instance, except of the skin about the genitals, where it was very striking. About the face, arms and neck, it was also sufficiently marked to at once attract attention.

The subject was considered an old man at the institution from which he came, but, with the exception of gray hair and baldness, there were none of the characteristics usually noticed in old subjects. The teeth were sound; the muscles remarkably well developed and free from fat; the skin was unwrinkled, and the subject was considered a remarkably good one. Death was said to have resulted from "old age and general debility;" there being no evidence of any disease.

On examination, the viscera were found sufficiently natural, and the supra-renal capsules healthy. The discoloration of the serous surfaces figured by Addison, were well shown upon the peritoneum; the mesentery, appendices epiploicæ of the sigmoid flexure, and some parts of the peritoneum covering the anterior wall of the abdomen, being well sprinkled with black specks not unlike "fly-blows"

JAN. 11th, 1858.—*Cancer of the Breast, from a nursing Woman.*—Dr. J. M. WARREN showed the specimen, which displayed the ducts filled with milk.

Dr. W. said that the patient labored under an accumulation of misfortunes seldom falling to the lot of one person. She had been confined to her bed for a fortnight, from the excessive pain and constitutional disturbance of the cancer. She was in a starving condition. She was nursing one child, and six months gone with another. Her husband was in a consumption; and the day after she entered the Hospital her eldest son was brought in with a fractured leg. The operation was, in a great measure, a palliative one, and thus far had afforded her entire relief. Some facts of this woman's history are interesting, and, at his request, Mr. Stickney, the surgical house pupil, had put them together.

Catherine McKenly, aged 43; is of large frame, and says she was strong and robust, and never had a sick day until the present time.—Had been in America 23 years. Came over from Ireland when 19 years old, and during her nineteenth year menstruated for the first time, the periods recurring regularly up to her first pregnancy. Was married four years after her arrival in this country, at the age of 23, and, in eleven months after, gave birth to twins. Had been married nineteen years in June next; had borne fifteen children, and is now six months advanced in pregnancy with her sixteenth.

The first pair of twins she nursed two and a half years, and, in about a week after, gave birth to another pair. These she nursed at once, and continued to do so for about the same length of time (two and a half years). Three of these children are now dead. The fourth, who was the most weakly, and of the first pair, is still alive, and now in Ward 28, on account of a fracture of the left leg.

She does not remember that she has ever, at any time, had her breasts entirely free from milk, since the first birth; and at this moment she has a small quantity in the left breast—having weaned her babe but two days before she entered the Hospital. Says she has invariably nursed each child till the birth of its successor. Does not recollect having menstruated but two or three times, in all, since being married.

Eleven children have died, and the four who are living are not very robust, probably from surrounding circumstances. Three died from hydrocephalus; one, from scalding with water; one from measles. The cause of death of the other six, she cannot state.

During all this time, she has obtained a living by days' work from home; frequently paying another woman twenty-five cents to care for her child till her return at night.

